# A Phylogeographic Assessment of the Malagasy Giant Chameleons (*Furcifer verrucosus* and *Furcifer oustaleti*)

**DOI:** 10.1371/journal.pone.0154144

**Published:** 2016-06-03

**Authors:** Antonia M. Florio, Christopher J. Raxworthy

**Affiliations:** Department of Herpetology, American Museum of Natural History, New York, NY, United States of America; National & Kapodistrian University of Athens, Faculty of Biology, GREECE

## Abstract

The Malagasy giant chameleons (*Furcifer oustaleti* and *Furcifer verrucosus*) are sister species that are both broadly distributed in Madagascar, and also endemic to the island. These species are also morphologically similar and, because of this, have been frequently misidentified in the field. Previous studies have suggested that cryptic species are nested within this chameleon group, and two subspecies have been described in *F*. *verrucosus*. In this study, we utilized a phylogeographic approach to assess genetic diversification within these chameleons. This was accomplished by (1) identifying clades within each species supported by both mitochondrial and nuclear DNA, (2) assessing divergence times between clades, and (3) testing for niche divergence or conservatism. We found that both *F*. *oustaleti* and *F*. *verrucosus* could be readily identified based on genetic data, and within each species, there are two well-supported clades. However, divergence times are not contemporary and spatial patterns are not congruent. Diversification within *F*. *verrucosus* occurred during the Plio-Pleistocene, and there is evidence for niche divergence between a southwestern and southeastern clade, in a region of Madagascar that shows no obvious landscape barriers to dispersal. Diversification in *F*. *oustaleti* occurred earlier in the Pliocene or Miocene, and niche conservatism is supported with two genetically distinct clades separated at the Sofia River in northwestern Madagascar. Divergence within *F*. *verrucosus* is most consistent with patterns expected from ecologically mediated speciation, whereas divergence in *F*. *oustaleti* most strongly matches the patterns expected from the riverine barrier hypothesis.

## Introduction

The chameleons *Furcifer oustaleti* (Malagasy giant chameleon) and *Furcifer verrucosus* (Warty chameleon) are both CITES species that are among the world’s largest chameleons. These two species are also morphologically similar and are often misidentified in the field, which has resulted in unclear species range limits. *F*. *oustaleti* and *F*. *verrucosus* are ideal species for a phylogeographic study because they are both endemic to Madagascar, broadly distributed, and previous studies indicate that they are found in partial sympatry. *F*. *oustaleti* and *F*. *verrucosus* are closely related based on both morphological and genetic data [1, 2], with the most recent studies reporting that they are sister species [3, 4, 5]. *F*. *oustaleti* has also been recently introduced and established in southern Florida, USA, and it is most probable that this has occurred through the pet trade [6].

The taxonomic history of *F*. *verrucosus* has been unstable for over 150 years, since first described in 1829 [7]. The species *Chamaeleon monilifer* was listed as a synonym of *F*. *verrucosus* [8] in 1831. Additionally, a new species, *Chamaeleo semicristatus*, from the southern tip of Madagascar, was differentiated from *F*. *verrucosus* with the following characters: flatter occipital region, strongly compressed rostral crest composed of large, conical tubercules, absence of a vertebral crest on the posterior part of the body, and a complete lack of a ventral crest [9]. *Ch*. *semicristatus* was later considered a synonym of *F*. *verrucosus* by Hillenius in 1959 [10], while another taxonomist, Mertens, gave this taxon subspecific rank (*Furcifer verrucosus semicristatus*) without explanation in 1966 [11]. Mertens described this subspecies as being widespread throughout the island, but most commonly found in the dry southwestern region. Brygoo [12] tentatively agreed with Hillenius [10], but proposed that *Ch*. *v*. *semicristatus* be retained as a subspecies.

*F*. *oustaleti* has always been considered as a single species since first described by Mocquard 1894 [13]. *F*. *oustaleti* was morphologically distinguished from *F*. *verrucosus* by head casque angle (the angle formed by the slit on the mouth and a straight line from the commissure of lips at the posterior end of the helmet is close to 90° in *F*. *oustaleti*, while it is >90° in *F*. *verrucosus)*, axillary pit presence, and tubercule development on the flanks. Brygoo [12] found that the most important character distinguishing these two species is the number of tubercules on the dorsal crest (≥ 45 in *F*. *oustaleti* and < 40 in *F*. *verrucosus*). Another study done by Bourgat and Brygoo [14] however did note hemipenial variation within both *F*. *oustaleti* and *F*. *verrucosus*, and Klaver and Böhme [15] also discussed karyological variation within both species.

Evaluating genetic divergence within widespread sister species, such *F*. *verrucosus* and *F*. *oustaleti*, is one way to identify the factors driving divergence in Madagascar. Recent research efforts have made substantial progress in our knowledge of speciation, but the processes driving diversification are still not well documented by empirical studies for many groups. Speciation is best studied by focusing on recent divergence events to more closely meet the assumption that the species geographic range has not changed over time [16]. However, setting species limits is often difficult in recently evolved groups due to low genetic variability and morphological crypsis [17, 18]. A potential solution to this problem is to utilize an integrative approach to incorporate multiple lines of evidence (e.g., morphology, genetics, and ecological niche modeling). This helps to strengthen species hypotheses and pinpoint the processes underlying diversification [19, 20, 21].

The island of Madagascar is a model system for studying genetic divergence and speciation [22]. The climate and geography, both current and past, of Madagascar has certainly influenced species distributions and patterns of divergence. The island has been isolated since about 88 MYA, and experienced a generally cooler and drier climate during the Pleistocene [23]. Presently, orographic uplift and trade winds create a general precipitation gradient from the humid northeastern and eastern rainforest to the dry southwestern deserts [24]. The extent to which these factors have contributed to diversification can be best studied utilizing a comparative approach in recently diverged groups that are currently undergoing population divergence and may, overtime, undergo speciation. This allows for an assessment of whether similar forces affect population processes across multiple species in the same geographic region, and from this information we can begin to draw inferences about the underlying causes of broad scale patterns among biodiversity [25]. Elucidating these patterns is important for all regions, including Madagascar, that have a high number of endemic species and exceptional species richness under continued anthropogenic threat [26].

Numerous studies have characterized patterns of diversification in Madagascar as a way to understand the rich species diversity found on the island. This has resulted in many diversification hypotheses [22, 27] that offer possible explanations for the production of this biodiversity. In this study, we focused on four hypotheses that we consider the most likely candidates for diversification, based on our chameleon target group distributions (e.g not montane) and ecology (see Table 1). Each of these hypotheses results in specific patterns that we use here to assess support between diversification models for our target chameleon group.

**Table 1:** Diversification models proposed for Madagascar and expected patterns.

Hypothesis	Predictions for Sister Species Distributions	Sister Species Ranges	Divergence Times between Sister Species	Sister Species Niches
**Watershed**	Allopatric	Adjacent Watersheds	Pleistocene	Divergent
**Ecologically Mediated**	Parapatric	Meet at Ecotones	No Constraint	Divergent
**Riverine Barrier**	Allopatric	Across rivers	River Formation	Similar
**Ecogeographic Constraint**	Allo- or Parapatric	East/West	No Constraint	Divergent

The watershed hypothesis [28] proposes that glacial periods caused species distributed in lower elevation watersheds to became trapped in arid pockets, and diversify in isolation. The hypothesis of ecologically mediated speciation [29, 30] proposes that the niches of sister species become divergent as they adapt to ecotones under disruptive selection and assortative mating. The riverine boundary hypothesis [31,32] proposes that the river systems in Madagascar have restricted gene flow leading to divergence between populations. Lastly, the ecogeographic constraint hypothesis [33] proposes that the abrupt transition in habitat between eastern and western Madagascar allows for initial east-west divergence within widely distributed species, with subsequent speciation constrained to within eastern and western regions. We specifically chose to test between these speciation hypotheses because several have shown to be potential drivers of divergence in chameleons (riverine boundary and ecogeographic constraint [4], as well as the watershed hypothesis [including *F*. *verrucosus* [34]), or in lizards more generally (ecologically mediated speciation [29, 30]).

## Methods

### Ethics statement for animal care and field sampling

Tricaine Methanesulfonate (MS222) was used for the euthanasia of reptiles and amphibians, which is approved by the Herpetological Animal Care and Use Committee (HACC) of the American Society of Ichthyologists and Herpetologists (http://www.asih.org/files/hacc-final.pdf), and the American Veterinary Medication Association (http://www.avma.org/issues/animal_welfare/euthanasia.pdf). First, the animal was anesthetized with 0.1–0.5 ml of 1% MS222 solution (buffered to a pH of 7.0–7.4 with sodium bicarbonate) injected into the coelomic cavity. After loss of righting reflex and lack of response to stimuli (e.g., a toe pinch), the animal was then euthanized with a 0.1–0.5 ml intracoelomic injection of 50% unbuffered MS222 solution. This procedure was followed by Conroy et al. [35], and also approved by the American Museum of Natural History IACUC committee.

Field studies in Madagascar were made possible due through the agreement of the Ministries des Eaux et Forêts, the Association Nationale pour la Gestion des Aires Protégés (ANGAP), and the Université d’Antananarivo, Département de Biologie Animale (especially D. Rakotondravony and H. Razafindraibe).

### Focal species sampling

A total of 129 individuals belonging to *Furcifer oustaleti* (n = 89) and *Furcifer verrucosus* (n = 40), collected between 1990 and 2012, were included for analysis. The closely related species *Furcifer labordi*, *Furcifer major*, and *Furcifer antimena* were included to test the sister species relationship of *F*. *verrucosus* and *F*. *oustaleti* [1, 2, 5]. *Furcifer campani* was used as the *Furcifer* outgroup taxon to root all phylogenetic trees. The following chameleon species were also included in the species tree analysis to estimate divergence dates: *Chamaeleo namaquensis*, *Chamaeleo chamaeleon*, *Furcifer cephalolepis*, and *Furcifer polleni*. These species are distantly related to the ingroup taxa, and were only included to allow for the fossil calibration in the divergence dating analysis.

In most cases, chameleons were collected during night surveys in the rainy season (approximately December through April) using headlamps to find individuals roosting on vegetation. Date, time, and longitude/latitude of each individual (using GPS, altimeter, or 1:100,000 topographic maps) were recorded at the time of collection. Voucher specimens were euthanized and fixed in 10% buffered formalin and then later transferred to 70% ethanol. Liver and/or thigh muscle was preserved in 95% ethanol or tissue buffer for later DNA extraction [36]. Voucher specimens and tissues are deposited at the American Museum of Natural History (AMNH), the University of Michigan Museum of Zoology (UMMZ), and the University of Antananarivo Department of Animal Biology (UADBA). Abbreviations for tissues samples are RAN (Ronald A. Nussbaum), RAX (Christopher J. Raxworthy), AF (Antonia M. Florio) MVZ (Museum of Vertebrate Zoology), and MCZF (Museum of Comparative Zoology). Localities, sample numbers, coordinates, and Genbank accession numbers for all samples are provided in S1 Table.

### Phylogeographic analyses

DNA was extracted from all tissue samples using the QIAGEN DNeasy Blood & Tissue kit (Valencia, CA) following the manufacturer’s instructions. Fragments for two mitochondrial genes (NADH dehydrogenase subunit 2 (ND2) and NADH dehydrogenase 4 (ND4)) and two nuclear genes (recombination activating gene-1 (Rag1) and oocyte maturation factor Mos protein gene (Cmos) were amplified. Polymerase chain reaction was carried out under locus-specific optimal annealing temperatures (see Table 2).

**Table 2:** Primer information for the genes utilized in this study. In most instances, genes were amplified with 35 repeated cycles (96°C for 1 min, locus-specific annealing T° for 1 min, and 72°C for 1 min).

Primer	Gene	Reference	Sequence	Annealing Temp. (°C)
ND2_oustF	ND2	This study	5’ TTATTACYGCCTCAAGCCACCACTG 3’	52
ND2_oustR	ND2	This study	5’ TTGGGGTRAANCCYGTTAGTGGTGG 3’	52
ND4	ND4	[37]	5' CACCTATGACTACCAAAAGCTCATGTAG 3'	54
LEU	LEU	[37]	5' CATTACTTTTACTTGGATTTGCACCA 3'	54
Rag1F	RAG1	[4]	5' GCCTCTCTRGACAAAGTCAGA 3'	52
Rag1R	RAG1	[4]	5' AGGATGTTCAGGAAGGATTTCAC 3'	52
CMOS3BradyF1	Cmos	[5]	5’ CCAGCCAAMGGTGGAAAGTTA 3’	52
CMOS17BradyR	Cmos	[5]	5’ TACTGCCGGTCCCCMAGATAAGG 3’	52

PCR products were cleaned using MultiScreen PCRμ96 Filter plates (Millipore, Billerica, MA, USA) and sequenced in both directions using BigDye v.3.0 (Applied Biosystems, Foster City, CA, USA) on an ABI 3730 automated DNA sequencer. Sequences were edited in GENEIOUS v.5.3.6 (Biomatters, Auckland, New Zealand). Multiple sequence alignments were generated using MUSCLE [38], with 1000 iterations and default gap opening cost of -1. Leading and lagging ends were trimmed to remove any missing data at the alignment edges. Haplotypes for nuclear sequences were inferred using PHASE v2.1 [39, 40] as implemented in DnaSP v5 [41]. Runs consisted of 1000 main iterations with an initial 100 iterations for burn-in and a thinning interval of 1.

Phylogenetic analyses of the mitochondrial data were conducted using maximum parsimony (MP), maximum likelihood (ML), and Bayesian inference (BI). MP was carried out with TNT v1.1 [42] and WINCLADA v1.0 [43] with equal weighing of all characters, and heuristic search option set at 500 random addition replicated using the New Technology Search option. Bootstrap support values were calculated for MP with 500 random addition replicates under a full heuristic search with 10 random addition sequences. ML was carried out in RAxML [44] with the RAxMLgui0.93 [45] using the ML + thorough bootstrap analysis option with 10 runs and 500 repetitions. Due to the large number of individuals included in the analysis and the low genetic divergence, the GTR+CAT algorithm was applied to analyze the data because it allows rapid navigation into a search space in which trees score well under GTR+ Γ but at significantly lower computational costs and memory consumption [44]. We also ran a combined mitochondrial and nuclear analysis using the same parameters to ensure that there was no conflict between the datasets.

The appropriate model of evolution for each gene was determined with JModelTestv0.1.1 [46, 47] by calculation of the highest Aikaike Information Criteria (AIC) value. Because only a limited number of models are available for use in both MrBayes and *BEAST, the more parameterized model was chosen and the appropriateness of this model was verified through visualization of transition and transversion rates in Tracer v1.7 [48]. Bayesian posterior probabilities were calculated using the Metropolis-coupled Markov chain Monte Carlo (MCMCMC) sampling approach in MrBayes v3.2.1 [49, 50]. BI searches consisted of one cold chain and three hot chains, and analyses were run for ten million generations with trees sampled every 1000 generations. The branch length prior was set to an unconstrained exponential with parameter 50.0 for a more accurate assessment of branch lengths because the datasets were composed of closely related individuals [51]. All searches started with random trees and uniform prior probabilities were assumed for all possible trees. Stationarity was assessed by checking the convergence of likelihood scores across two runs using TRACER v1.4 [52] and with Are We There Yet? (AWTY–[53]) using AWTY online [54]. The first 20% of trees were discarded as “burn-in”, and the remaining trees were combined to form a 50% majority rule consensus tree and to determine nodal posterior probabilities.

Nuclear data were analyzed independently from mitochondrial data in two ways. First, SPLITSTREE v4.12.13 [55] was used to identify identical haplotypes and to reconstruct haplotype median-joining networks for each nuclear locus. Median-joining was used to analyze each nuclear gene separately. Second, we assessed the number of genetic groups, without bias from existing species designations or geographical distribution, using the model–based clustering algorithm as implemented in the program STRUCTURE v2.3.4 [56]. An admixture model was assumed, and the burn-in length was set at 10^5^ steps, followed by 5x10^6^ MCMC iterations. The number of potential groups was set to very between K = 1 to K = 10, and 10 repetitions were carried out for each value of k to ensure consistency in probability estimates. We evaluated the appropriate number of clusters in two ways: (1) by averaging the log probability of the data (Pr(X|K)) for each K and choosing the lowest value, and (2) by using the ΔK method [57]. We estimated k with the nuclear and mitochondrial data together, and with the nuclear data alone.

### Species tree reconstruction and divergence dating

Strongly supported mitochondrial clades that also show evidence of differentiation with median-joining networks of either nuclear gene and genetic clustering with STRUCTURE were considered as potential species in *BEAST v1.7.4 [48]. The statistic *d*_*XY*_ [58] was used to measure the mean number of nucleotide substitutions between potential species. In addition, a molecular clock test was performed by comparing the maximum likelihood value (likelihood ratio test) for the given topology with and without the molecular clock constraints [59] in MEGA5.1 [60]. The two-mitochondrial genes were linked to form a single gene tree and a Yule Process prior was placed on all gene trees. Default priors were used for all analyses, and a uniform prior between 0 and 100 was set for the ND4 and CMOS clock rates as well as the ucld.mean parameter for RAG1. The species tree analyses was run for 200 million generations and trees were sampled every 10,000 generations, with the first 20% of sampled trees discarded as burn-in.

Divergence time estimates were assessed in the following two ways: (A) using the following calibration: minimum age for genus *Chamaeleo* (18 MYA based on the age of the fossil *Chamaeleo andrusovi* [61, 62] and (B) using the previously published molecular rate (0.65% change per lineage per million years) for ND2 [63, 64, 65]. Rates were estimated for all other gene partitions (ND4, RAG1, and CMOS). The *Chamaeleo* fossil was assigned using a lognormal prior and the standard deviation around the mean (18 MYA) was set at 0.13 to allow variance to encompass the lower part of the Miocene. All species tree analyses were run for 400 million generations and trees were sampled every 40,000 generations, with the first 20% of sampled trees discarded as burn-in.

### Ecological niche modeling

Ecological niche models were constructed and tests of niche similarity or divergence were performed to help differentiate between diversification models (see Table 1; for example: conserved niches between sister groups would be consistent with the watershed and riverine barrier hypotheses). After deletion of duplicate records, 134 unique localities were included for the development of all ecological niche models (ENMs). Climate data was taken from the WorldClim database (http://worldclim.org/) [66], with the 19 bioclimatic variables used for ENM analyses in Maxent v3.3.3k [67]. All occurrence localities and environmental variables were resampled to an oblique Mercator projection at 1 km^2^ resolution [68] using ARCMAP [69]. Default values were used for the maximum number of iterations (500) and for the convergence threshold (10^−5^). The minimum training presence (or lowest predicated value (LPT) of environmental suitability) was chosen for each model as the decision threshold. The ENM was visualized in ARCMAP by reclassifying the continuous data to create a binary prediction, and all values above the LPT were reclassified as suitable environment.

### Tests of niche conservatism or divergence

Niche conservatism or divergence between potential species was tested using the program ENMTools v1.3 [70]. Niche overlap values were first calculated using the Schoener’s D metric [71] in ENMTools [72]. The niche identity test was used to test whether the niches between sister clades are identical. We also implemented the background similarity test since it is unlikely that allopatric species will have identical niches as they probably do not have the same environmental conditions available to them [73]. This test differs from the niche identity test by testing whether the niches of sister clades are more or less similar than expected based on the background environment in which they occur. The background region for each species was defined by creating a minimum convex polygon around the known locality points and then extracting 1000 random coordinates; this was accomplished using Hawth’s Tools [74] in ArcGIS. The number of background points used out of the 1000 random coordinates was equivalent to the number of localities available for the clade from which the random points were drawn [75]. For example, if clade A had 25 points and clade B had 50, then the 25 points from clade A were compared to 50 random background points from the distribution of species B. The analyses were run with 500 replicates and results were visualized as histograms using R [76].

### Morphological measurements

Morphological measurements were included to assess whether any genetic patterns were coincident with morphological variation. The morphology of the specimens were described using standard morphological terms and methods [10, 36]. This included snout-vent-length (SVL), number of dorsal crest cones (the number of raised scales (spine-like) found on the center of the back) and gular crest cones (row of small spines running down the center of the throat), and both the presence of axillary pits (pockets or depressions found under the front limbs of some lizard species) as well as enlarged tubercules (scales) on the flanks of the specimen. Measurements were analyzed separately for each sex so that sexual dimorphism could be assessed in each species. 24 *F*. *verrucosus* individuals (females = 9; males = 15) and 35 *F*. *oustaleti* individuals (females = 17; males = 18) were included in the analysis. Adults were defined as exceeding 100 mm in SVL, and chameleons sexed based on the presence of everted hemipenes (males) or the presence/absence of hemipenal bulges at the tail base.

## Results

### Phylogenetic and phylogeographic analysis

All 129 individuals of *F*. *oustaleti* and *F*. *verrucosus* were amplified for 4 loci (303 bp of ND2, 647 bp of ND4, 726 bp of RAG1, and 611 bp of CMOS). Downloaded sequences from Genbank were used to supplement some genetic data for the outgroup taxa in the divergence dating analyses (see S1 Table). JModelTest recovered the following models with the highest AIC for each gene: K80+G for ND2 and CMOS, K80+G for RAG1, and GTR+G for ND2 and ND4.

**Fig 1:**
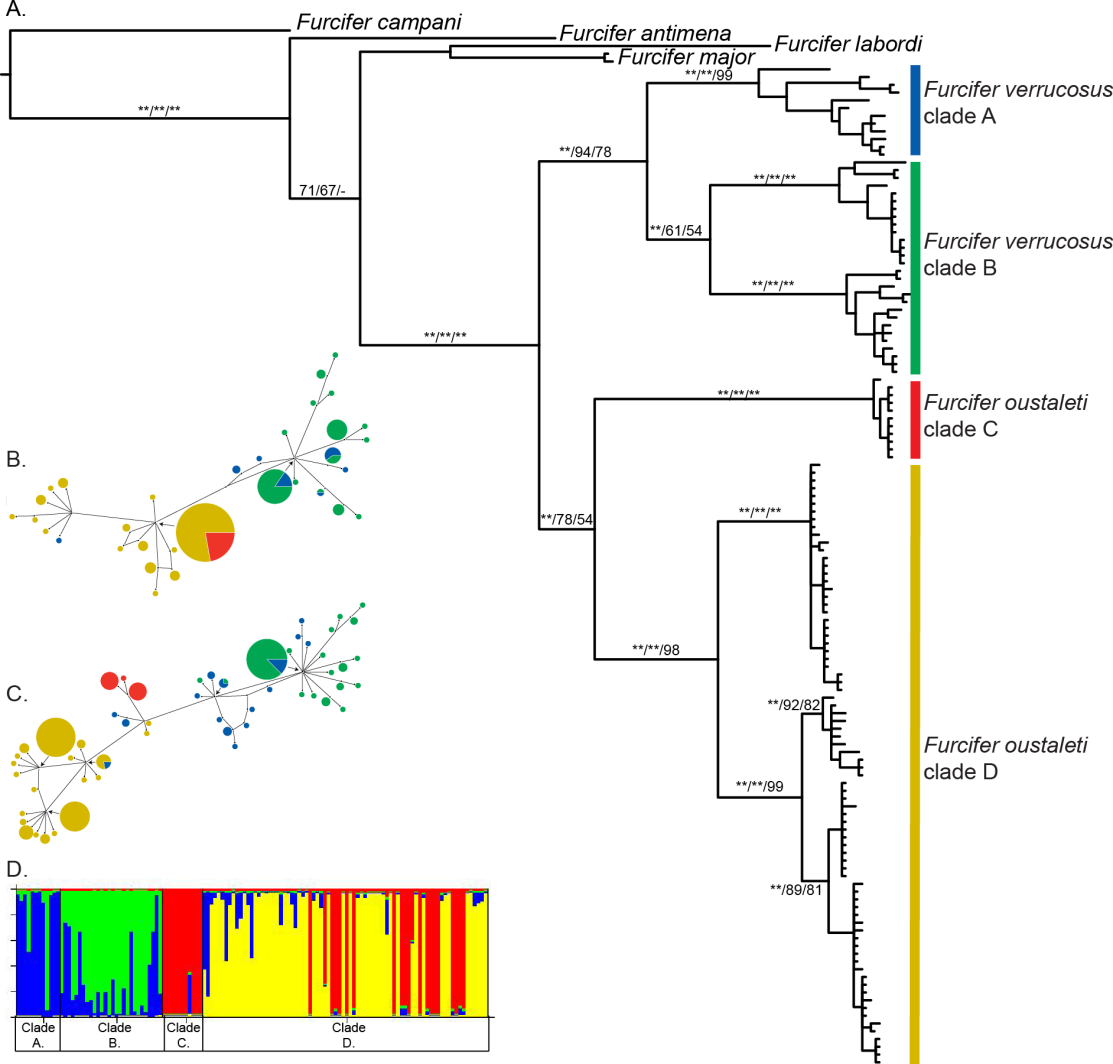
Genetic divergence within the *F*. *oustaleti* and *F*. *verrucosus* species complexes. (a) Phylogenetic relationships between the *Furcifer verrucosus* complex, the *Furcifer oustaleti* complex, and near outgroups using partial fragments of the mitochondrial genes ND2 and ND4, reconstructed on the ML tree. (BI/ML/MP; ** = 100%). Only groups with nuclear support are labeled as “clades” on the tree. (b–c) The median-joining haplotype networks for the nuclear gene CMOS (b) and RAG1 (c) also recover support for the *Furcifer verrucosus* complex and the *Furcifer oustaleti* complex with some differentiation within each species. (d) Results from the structure analysis including both the mitochondrial and nuclear data when k = 4. Mitochondrial clades with nuclear support are labeled A-D on the mitochondrial tree.

The topology of the mitochondrial tree (Fig 1A) is congruent across BI, ML, and MP, and support values are above branches (BI posterior support/ML bootstrap/MP bootstrap). The sister species relationship between *F*. *oustaleti* and *F*. *verrucosus* is strongly supported with all analyses. Each species is also strongly supported as monophyletic using all optimality criteria (BI, ML, and MP), with the exception of *F*. *oustaleti* with MP (*F*. *verrucosus* and *F*. *oustaleti* (see Fig 1A). Full sample names on the ML tree recovered using mitochondrial DNA are provided in S1 Fig. The combined dataset (mitochondrial and nuclear DNA) recovered a result identical to the mitochondrial tree (see S2 Fig).

Substantial genetic structure is recovered within both *F*. *oustaleti* and *F*. *verrucosus* using mitochondrial data (Fig 1A). Within *F*. *verrucosus*, three clades are well supported across all optimality criteria. Individuals confined to the southeastern region (clade A) are sister to all other *F*. *verrucosus* (clade B). Within *F*. *oustaleti*, there are five strongly supported monophyletic clades recovered with mitochondrial data. This includes a deeply divergent clade (clade C) confined to the northernmost regions of the island, that is sister to all other *F*. *oustaleti* samples (clade D). The genetic structure within clade D is partially geographically structured with more southern individuals separate from northern groups within the clade.

Less genetic structure is recovered when the phased nuclear genes are analyzed using a median-joining network. With CMOS, *F*. *oustaleti* and *F*. *verrcusosus* are recovered as distinct genetic clusters, but other mitochondrial clades are not distinct (Fig 1B). In addition, one haplotype from a single *F*. *verrucosus* individual falls within *F*. *oustaleti*. There is some evidence for genetic substructure in *F*. *oustaleti* but this genetic division does not correspond with either the mitochondrial clades or with geography. With RAG1, there is some support for two genetic clades in *F*. *verrucosus* and two clades within *F*. *oustaleti* that also correspond with the results from the phylogenetic analysis of the mitochondrial data (Fig 1C). Similar to the CMOS data, there are a few exceptions to this general pattern. For example, one haplotype from two *F*. *verrucosus* individuals is identical to a haplotype found in several *F*. *oustaleti* samples.

With the program STRUCTURE v2.3.4, the lowest Pr(X|K) and the largest ΔK were found for K = 4 (Pr(X|K) = -1378.75; Fig 1D) when nuclear and mitochondrial data were analyzed together. We then assessed the number of genetic clusters with the nuclear data alone to make sure the mitochondrial data was not driving the STRUCTURE analysis, and the lowest Pr(X|K) was also found when K = 4 (Pr(X|K) = -1072.23), but the largest ΔK (or greatest improvement in likelihood) occurs when K = 2. Mitochondrial divergence between clades is high, with d_XY_ values ranging from 8.4 to 11.8% (see Table 3). Highly–supported mitochondrial clades with some evidence of nuclear differentiation were considered as potential cryptic species in all subsequent analyses, and are labeled on Fig 1A.

**Table 3:** *d*_*XY*_ values estimated between clades in *F*. *verrucosus* and *F*. *oustaleti*. Values below the gray are estimated from the gene ND2, while those above the gray are those estimated from the gene ND4.

	A	B	C	D
**A**	-	0.084	0.107	0.115
**B**	0.088	-	0.110	0.116
**C**	0.114	0.118	-	0.107
**D**	0.097	0.118	0.101	-

#### Species tree analysis and divergence dating

**Fig 2:**
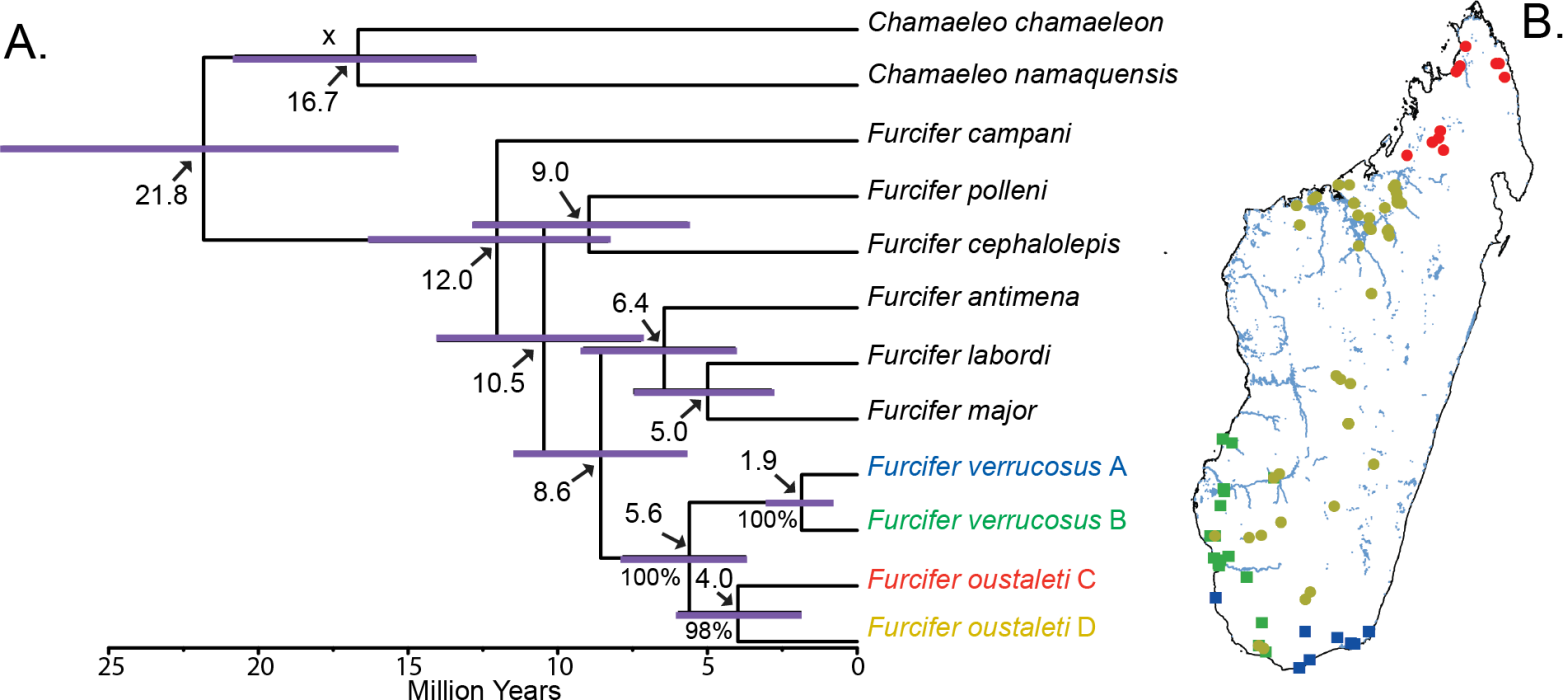
Species tree results and geographic distribution for the *F*. *verrucosus* and *F*. *oustaleti* species complexes. (a) Species tree analysis with divergence dating using the fossil calibration *Ch*. *andrusovi* (X). All divergence times are indicated with an arrow. Divergence between clades of *F*. *verrucosus* occurred approximately 1.9 MYA, while divergence between clades of *F*. *oustaleti* occurred earlier, approximately 4.0. MYA. The 95% confidence intervals are indicated by purple bars placed over nodes. The monophyly of *F*. *verrucosus* and *F*. *oustaleti*, and the clades within each species, are also well supported with high posterior probabilities (values below branches). (b) The geographic distribution of individuals within clades A-D. There is some sympatry between *F*. *verrucosus* and *F*. *oustaleti* in southern and southwestern Madagascar.

The null hypothesis of equal evolutionary rates was not rejected for ND2, ND4, and CMOS (p>0.05), but was rejected for RAG1 (p<0.05). Therefore, a strict molecular clock was applied to the ND2, ND4, and CMOS gene partitions in *BEAST, and a lognormal uncorrelated relaxed clock was applied to the RAG1 partition. The sister species relationship of the *F*. *verrucosus* and *F*. *oustaleti* complexes is recovered with high posterior support in the species tree (100%), as well as the sister species relationships of species A–B and C–D within each complex (100% and 98%, respectively–see Fig 2A).

We first assessed divergence dates using one calibration: a fossil constraint on the age of the genus *Chamaeleo* and the results of this analysis are shown in Fig 2A. Based on these dates, divergence between the *F*. *oustaleti* and *F*. *verrucosus* complexes is estimated at 5.6 MYA (3.7–7.8 MYA [95% confidence intervals]). These results indicate that the *F*. *oustaleti* and *F*. *verrucosus* complexes diverged during the Pliocene or the upper Miocene. The divergence date between clades C and D within the *F*. *oustaleti* complex is estimated at 4.0 MYA (1.8–6.0 MYA), while the clades A and B within the *F*. *verrucosus* complex are estimated to have diverged only 1.9 MYA (0.9–3.0 MYA). This analysis dates the divergence within the *F*. *oustaleti* complex as occurring at the end of the upper Miocene or during the Pliocene, whereas the divergence in the *F*. *verrucosus* complex occurred either at the end of the Pliocene or during the Pleistocene. Additionally, the age of *Chamaeleo* was recovered as 16.7 myr (12.8–20.8 MYA).

For comparison, we also assessed divergence dates using the ND2 rate of (0.65% change per lineage per million years–species tree results with divergence dating using this rate is available in S3 Fig). With this rate, divergence between the *F*. *oustaleti* and *F*. *verrucosus* complexes is estimated at 8.5 MYA (6.6–10.4 MYA [95% confidence intervals]). The divergence date between clades C and D is estimated at 6.2 (3.5–8.4 MYA), while clades A and B diverged only 2.6 MYA (1.5–4.2 MYA). These dates are all older than those inferred from the fossil constraint analysis. However, this does not change the geologic period where divergence occurred as outline above. In addition, the dating analysis using the ND2 rate alone recovered the age of *Chamaeleo* as 24.5 MYA (18.7–30.6 MYA–S3 Fig).

### Distribution patterns and ecological niche modeling

**Fig 3:**
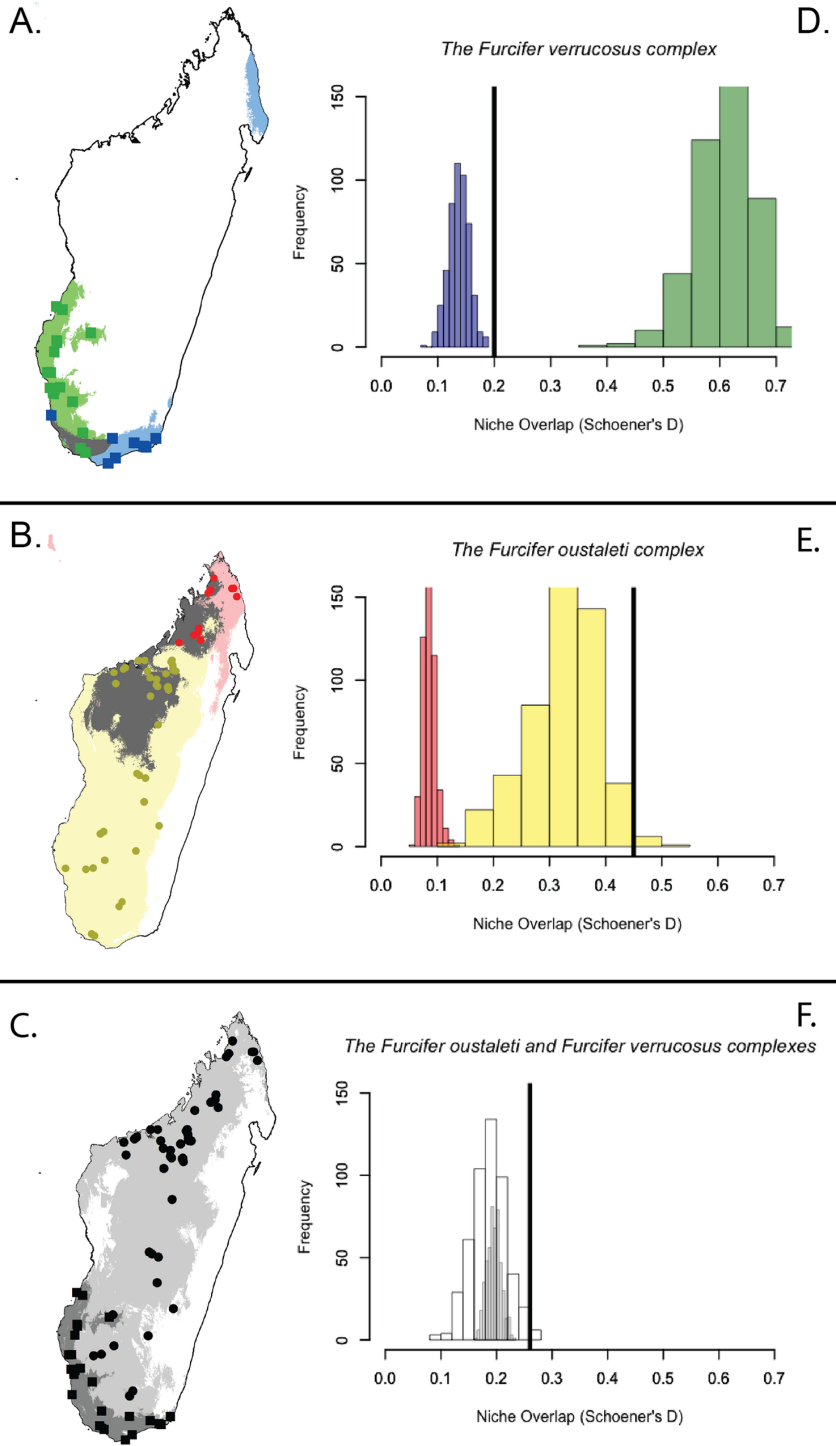
Ecological niche models and background tests of niche divergence and conservatism. **Any predicted overlap is ENMs is indicated by dark gray.** (a–c) Projected ENMs created using Maxent for clades A and B within the *F*. *verrucosus* complex (a), clades C and D within the *F*. *oustaleti* complex (b), and for each the *F*. *verrucosus* complex (dark gray) and the *F*. *oustaleti* complex (c). (d–f) The results of the niche background test implemented in ENMTools are shown in Fig 3d – 3f, with the Schoener’s’s D value for the species of interest indicated by a black line through the x-axis (the x-axis represents the distribution of pseuodreplicates). Niche divergence is supported for clade B based on a null background distribution of clade A,but that niche conservatism is supported in the reverse case for clade A (d), niche conservatism is supported for clades C and D within the *F*. *oustaleti* complex (e), and niche conservatism is supported for the *F*. *oustaleti* complex and the *F*. *verrucosus* complex (histogram without color–f).

The range of *F*. *verrucosus* is confined to the extreme south and southwest of Madagascar, whereas *F*. *oustaleti* is distributed throughout the island and sympatric with *F*. *verrucosus* in the south and southwest (see Fig 2B). Ecological niche models for each species are shown in Fig 3A–3C. The predicted niche of clade A is mostly southeastern Madagascar, but there is an area of over-prediction in northeastern Madagascar where *F*. *verrucosus* is not known to occur (Fig 3A–note that one individual (RAX 11194) in species A was not included ENM analyses because it represents a translocated individual. In contrast, the predicted niche for clade B is recovered as southwestern and southern Madagascar, and is mostly constrained to the coast. The gray areas on the map represent areas of niche overlap between the two species.

Ecological niche models for the species within the *F*. *oustaleti* complex show a very different pattern (Fig 3B). The niche for clade C is predicted only in the very north and northwest of the island. However the niche for clade D is predicted to be almost all of Madagascar (see yellow on Fig 3B), except for the very north and the entire east of the island. There is substantial amount of niche overlap between the two species.

We also created ecological niche models for the *F*. *verrucosus* complex and *F*. *oustaleti complex*, individually (Fig 3C). The niche for the *F*. *oustaleti* complex is indicated by light gray and covers almost all of the island of Madagascar. The niche for the *F*. *verrucosus* complex is instead confined to the very southern region of Madagascar, and is entirely encompassed by the predicted niche of the *F*. *oustaleti* complex (and is therefore shown in dark gray on Fig 3C).

### Tests of niche conservatism and divergence

Niche identity was rejected for all comparisons (clades A vs. B; clades C vs. D; and the *F*. *oustaleti* complex vs. the *F*. *verrucosus* complex–see S2 Table) because Schoener’s D was lower than the randomized distribution of the 500 pseudoreplicates. The results of the niche background test implemented in ENMTools are shown in Fig 3D–3F, with the Schoener’s D value (calculated from the ecological niche models created from the actual occurrences) indicated by a black line through the x-axis. The niche of clade A is more similar than expected by chance when compared to the background of clade B (Fig 3D). In contrast, the niche of clade B is more different than expected by chance when compared to the background of clade A. Within the *F*. *oustaleti* complex, both clade C and clade D have niches than are more similar than expected by chance when compared to one another (Fig 3E). This pattern is also supported when comparing the *F*. *verrucosus* complex to the *F*. *verrucosus* complex, with niche overlap higher than expected by chance alone (Fig 3F). All values for the background test are provided in S3 Table.

### Morphology

**Fig 4:**
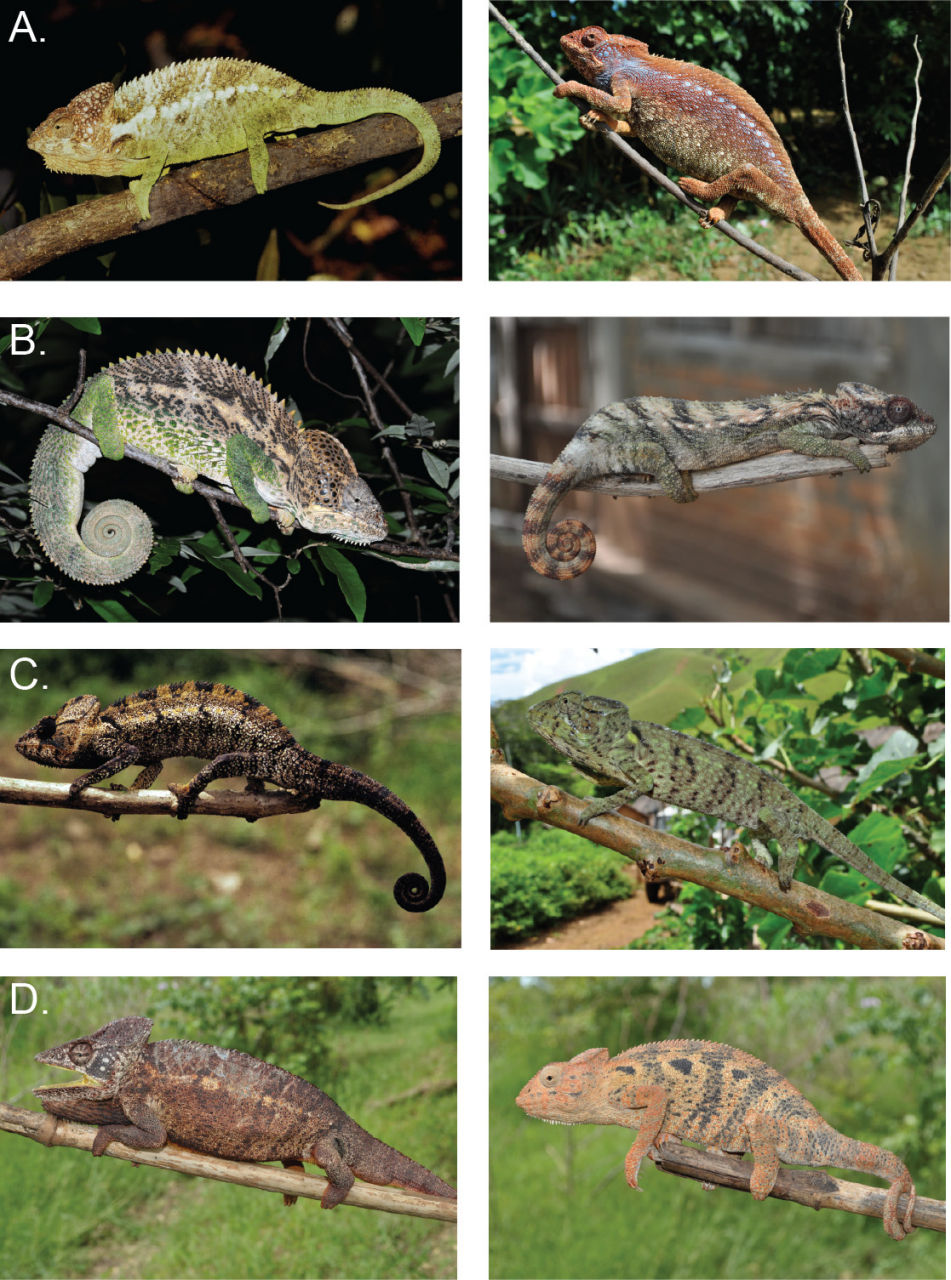
Live adult representatives for each species in the *F*. *verrucosus* and *F*. *oustaleti* complexes. **Letters correspond to clades on all other figures in this paper; males (m) on left and females (F) on right.** (a) *F*. *verrucosus* clade A (m: Fort Dauphin; f: Tsiaroa-Ampasy), (b) *F*. *verrucosus* clade B (m: Mangoky River; f: Ankatsakantsa Sud), (c) *F*. *oustaleti* clade C (m: Ambanja; f: Anoalakely), (d) *F*. *oustaleti* clade D (m and f: Mahabibo).

The morphological measurements and characters scored for the *F*. *verrucosus* and *F*. *oustaleti* species complexes are summarized in Table 4. *F*. *oustaleti* and *F*. *verrusosus* are distinguished by the number of dorsal cones in both females and males (>50 in *F*. *oustaleti*; <50 in *F*. *verrucosus*). This result confirms original findings that the number of dorsal cones distinguishes the species *F*. *oustaleti* from *F*. *verrucosus* [12]. The difference in dorsal cone number is especially striking for females (maximum dorsal cones is 13 in *F*. *verrucosus* compared to 76 in *F*. *oustaleti*). *F*. *oustaleti* individuals (maximum SVL = 261 mm/199 mm (males/females) also tend to be larger than *F*. *verrucosus* individuals (maximum SVL = 196 mm/138 mm). Representative individuals are provided in Fig 4. Based on the characters measured and scored, there are no clear morphological distinctions between the genetic clades (potential cryptic species) found within each species.

**Table 4:** Morphological variation in *Furcifer oustaleti* and *Furcifer verrucosus*.

Character	*Furcifer* species			
	*F*. *verr-A* (f = 2; m = 4)	*F*. *verr-B* (f = 7;m = 11)	*F*. *oust-C* (f = 3;m = 5)	*F*. *oust-D* (f = 14;m = = 13)
Maximum male SVL	164	196	222	261
Maximum female SVL	138	135	176	199
Male dorsal cones	31–44	30–42	68–74	54–70
Female dorsal cones	2	3–13	72–76	55–69
Male gular crest cones	12–18	12–21	14–25	14–21
Female gular crest cones	10–12	10–18	13–30	15–20
Axillary pits	-	-	+/-	+/-
Enlarged round tubercules on flanks	+	+	+/-	+/-
Body color	mostly green	green	brown or green	brown

All measurements in mm. Coloration is based on live animals at rest. Number of female and male specimen are shown below the clade name, and indicated by (f) and (m).

## Discussion

### Phylogenetic analyses

The *F*. *oustaleti* and *F*. *verrucosus* complexes are well supported as sister clades with mitochondrial data, and as distinct nuclear clusters with both nuclear genes analyzed. While there is substantial genetic variation recovered with mitochondrial data, there is little variation within each species complex with the nuclear genes. This is not surprising, since the two nuclear genes used in this study, CMOS and RAG1, have been shown to have low variation [77]. Therefore, the lack of phylogenetic structure found with these nuclear loci is most likely due to the nuclear loci acting as a lagging indicator of lineage divergence [78].

Within both species, the mitochondrial clades are geographically structured on the landscape of Madagascar (see Fig 3). Genetic structure in the *F*. *oustaleti* complex displays a north–to–south pattern in Madagascar. This is in contrast to the pattern recovered within the *F*. *verrucosus* complex, which shows a southwest–to–southeast pattern. Most interesting is that although three mitochondrial clades are recovered within the *F*. *verrucosus* complex, all southern individuals of the *F*. *oustaleti* complex form a single clade in the area of sympatry.

There are a few exceptions to the pattern described above, regarding the four distinct clades (A-D) with non-overlapping geographic ranges. One individual (RAX 11194) in clade A (mitochondrial and nuclear data) is located within the geographic range of clade B (see Fig 2B). This is most likely a case of accidental human mediated dispersal. Additionally, one haplotype of the individual (RAX 11299) falls within haplotypes of the *F*. *oustaleti* complex for the nuclear gene CMOS, but this individual is supported as *F*. *verrucosus* with mitochondrial DNA and the nuclear gene RAG1. Two other individuals (RAX 11943 and RAX 11949) both have RAG1 haplotypes identical to ones found in *F*. *oustaleti*, but are recovered as *F*. *verrucosus* with mitochondrial DNA and the nuclear gene *CMOS*. Assessing whether these are cases of incomplete lineage sorting or hybridization is beyond the scope of this study, but it is interesting to note that all the mixed haplotypes individuals occur in regions where the two species ranges are in close proximity, as would be the case if hybridization were occurring. Unfortunately, these individuals (RAX 11194, RAX 11299, RAX 11943, and RAX 11949) were not available for morphological analysis (either because they were juveniles or no voucher was taken), but it would be interesting in future studies to test any genetic exceptions with morphological identification.

### Divergence time estimation

Divergence time estimation requires a number of assumptions either about the rate of molecular evolution, or the time and placement of fossils [79, 80, 81]. To help alleviate this, we estimated chameleon divergence both ways and then assessed concordance. While the dates inferred using the ND2 molecular rate were generally older, the dates did not largely vary between analyses with respect to geologic period (see Fig 2 and S3 Fig). Additionally, one way to assess the validity of the dates inferred from a molecular rate is to see if the inferred dates are consistent with those obtained from the fossil record or biogeographical studies [82]. Using the rate from ND2 (0.65%) alone (see S3 Fig), we recovered dates that are consistent with previously documented chameleon fossils. For instance, the age of the genus *Chameleo* was recovered as 24.5 MYA (18.7–30.6 MYA) with the ND2 rate alone analysis, and this is consistent with fossil dates for the minimum age of *Chamaeleo* (*Chamaeleo andrusovi*– 18 MYA). The ND2 rate analysis (see S3 Fig) also estimated the origin of *F*. *cephalolepis* at 13.8 MYA (10.0–17.1 MYA) which does not contradict the biogeographic dates that the places the maximum age for *F*. *cephalolepis* and *F*. *polleni* at 12.5 MYA [83, 84, 85] (when considering the 95% confidence intervals). Interestingly, when we applied both the fossil calibration and this biogeographic calibration to the dating analysis (using a uniform prior (lower = 0; maximum = 12.5) so that the only constraint was on a maximum age of *F*. *cephalolepis*), we recover very similar results in regards to estimates of divergence dates for the groups (see S4 Fig).

It is important to note that the mean dates recovered by this study using a single fossil calibration (*Chamaeleo andrusovi*) are about half as old as those recovered with a larger dataset and additional calibrations in a 2013 study by Tolley et al. [5], even when considering the 95% confidence intervals. For example using the fossil calibration analysis, our mean divergence estimate for the age of *Chameleo* is 16.7 MYA, while this date was previously reported as occurring around 40 MYA. Additionally we found that *F*. *verrucosus* and *F*. *oustaleti* split 5.5 MYA, and Tolley et al., [5] found the split occurred around 10 MYA.

### Species distributions and ecological niche modeling

This is the first study of the Malagasy giant chameleons with comprehensive sampling corroborated with molecular data, which is especially important because there is substantial overlap in diagnostic morphological characters between *F*. *oustaleti* and *F*. *verrucosus* (as evidenced by the morphological results in Table 4). We have clarified the range of these chameleon species. From the point locality data alone, we found that the range of the *F*. *verrucosus* complex is restricted to southern and southwestern Madagascar, and the species complex is found only as far north as the Mangoky River in the southwest. However, the *F*. *oustaleti* complex has a large distribution, ranging as far south as Marolinta and throughout central and northern Madagascar. These distribution results of this study contrasts with past studies, especially regarding the distribution of *F*. *verrucosus*. While Hillenius [10] documented both species as distributed throughout Madagascar, but described the “center of the distribution” (or primary distribution) for *F*. *verrucosus* as south and southwestern, and that of *F*. *oustaleti* as more northern and eastern. Brygoo [12] reported *F*. *verrucosus verrucosus* from many regions on the island except the east coast and the northeast, but *F*. *verrucosus semicristatus* only from southern Madagascar. It is now apparent that while *F*. *oustaleti* is found throughout Madagascar, *F*. *verrucosus* is restricted to mostly southern and southwestern Madagascar.

Additionally, the two clades within the *F*. *verrucosus* complex have disjunct distributions, with clade A distributed almost solely in southeastern Madagascar, and clade B distributed in the south and southwest. Within the *F*. *oustaleti* complex, clades C and D also have disjunct distributions, with no apparent sympatry of ranges. The results of this study are consistent with a pattern of range overlap between older species divergences through post-speciational range changes (because there is geographic overlap between individuals in the *F*. *oustaleti* complex and the *F*. *verrucosus* complex), but little overlap and asymmetrical ranges between more recent divergences (as evidenced by the lack of syntopic distributions between clades A/B and clades C/ D) [86].

We used environmental niche models to evaluate the ecological tolerances of each species to visualize the extent of niche differentiation. Within the *F*. *verrucosus* complex, niche overlap between clades A and B is restricted to a narrow region (see Fig 3A). In contrast, within the *F*. *oustaleti* complex, the two clades have a large area of niche overlap in northwestern and northern Madagascar (see Fig 3B). This is because of a large area of over-prediction for clade C that reaches down into northwestern Madagascar and because the niche of clade D is predicted for the entirety of the island, except the eastern region. The poor performance of these models may reflect the high genetic differentiation (with mitochondrial data) of clades within clades D, and may indicate the existence of potential species within clade D, as previous studies have found that ecological niche models made with cryptic species lumped together generally give poor models [29].

Although niche overlap provides some information about niche differentiation, we complemented this approach by utilizing a background randomization procedure. One potential problem with the background analysis is defining the background region. The background area should ideally include the entire distribution of the sister species and possible areas of dispersal [77, 87]. To best fit these criteria, we constructed a minimum convex polygon around all known locality points [72].

Niche conservatism is supported both for the species within the *F*. *oustaleti* complex and for the *F*. *oustaleti* compared to the *F*. *verrucosus* complexes (Fig 3A–3F). The answer is less clear when interpreting the results for clades A and B within the *F*. *verrucosus* complex. Niche divergence is supported when clade B and is compared to the null background of clade A in the *F*. *verrucosus* complex, but niche conservatism is supported when clade B is compared to the background of clade A. This may be due to differences in the heterogeneity of the background, and because clades A and B both prefer an environment that is unavailable to species B [75, 88]. Consistent with results reported here, other studies found that the *F*. *verrucosus* complex was coincident with climate clusters, suggesting that climate may play a role in diversification in this group [34].

### Contrasting divergence patterns with the *F*. *lateralis* complex

The *F*. *lateralis* complex (along with *F*. *labordi*) includes *F*. *lateralis*, *F*. *viridis*, and *F*. *major*. These species are closely related to both the *F*. *oustaleti* and *F*. *verrucosus* complexes [1,2], and also occupy the same geographic area of Madagascar [4]. Based on results from a previous study [4], spatial patterns between the species within the *F*. *lateralis* complex, and both the *F*. *verrucosus* and *F*. *oustaleti* complexes are not congruent. For instance, the geographic split between *F*. *major* and *F*. *viridis* occurs in southern Madagascar, but further north (at the Mangoky River) than the split between clades A/B in the *F*. *verrucosus* complex. Additionally, there is no evidence of a split in northern Madagascar in the *F*. *lateralis* complex, as is found in the *F*. *oustaleti* complex. Unfortunately because of largely overlapping 95% confidence bars on the divergence between *F*. *labordi* and *F*. *major* (see Fig 2A), it is currently unclear whether divergence times between the groups are contemporaneous between these groups.

### What is driving divergence in Malagasy giant chameleons?

The phylogenetic results and distribution patterns, along with divergence time estimates and niche divergence/conservatism results, can be used to infer potential drivers of diversification in *F*. *oustaleti* and *F*. *verrucosus*. This is because expected patterns differ between the diversification models (watershed, ecologically mediated, riverine barrier, and ecogeographic constraint–see Table 1). The watershed hypothesis is not consistent within either species complex since sister species do not appear distributed in adjacent watersheds [28]. In addition, the ecogeographic constraint divergence model proposes that sister species distributions would occur in eastern and western Madagascar, which is not a pattern shown by either species complex [33]. Divergence within the *F*. *oustaleti* complex is best supported by the riverine barrier hypothesis [31, 32] because the two clades are allopatrically distributed (as determined by the point locality distribution) across the Sofia River and niches between sister clades are similar (conserved). In contrast, divergence within the *F*. *verrucosus* complex best fits the hypothesis of ecologically mediated speciation [29, 30] because the species are parapatrically distributed, the niche of *F*. *verrucosus* clade B has diverged with respect to clade A (see Table 5).

**Table 5:** Hypotheses supported as the driver of diversification between clades found within *F*. *verrucosus* and *F*. *oustaleti*.

Species	Hypothesis	Distributions	Ranges	Divergence Times	Niches
*F*. *verrucosus* A vs. B	Ecologically Mediated	Parapatric	Not tested in this study	Plio-Pleistocene	Divergent
*F*. *oustaleti* C vs. D	Riverine Barrier	Allopatric	Across rivers	Pliocene/ Miocene	Similar

It is not unexpected that two different modes of divergence are supported within these sister groups since recent studies have shown multiple drivers of speciation in Madagascar [27, 89, 90, 91]. It is interesting to note that the distributions of clades within *F*. *oustaleti* are coincident with the Sofia River. The Sofia River is a major river drainage in northwestern Madagascar that maintains water year-round. Although the Sofia River was not historically proposed as a potential major physical barrier to dispersal for other groups [92], it has been suggested to limit the global distribution of lemurs species in the north and south of Madagascar [93, 94, 95]. This study provides some support that this drainage is acting as a barrier to dispersal to individuals within the *F*. *oustaleti* complex and may be driving divergence between the two clades. There is currently no information on when the Sofia river formed. If it is found that the Sofia River formed during the Pliocene or Miocene (the divergence date found in this study for clades within *F*. *oustaleti*), this would further support this river as a driver of diversification in this group. There are other large rivers south of the Sofia River, that do not seem to be driving divergence within *F*. *oustaleti*, and it is currently uncertain why this is the case. In contrast to support the river barrier hypothesis, clades within the *F*. *verrucosus* best show patterns associated with ecologically mediated speciation. While ecologically mediated speciation can occur at any time period, it is interesting to note that divergence in the *F*. *verrucosus* dates to the Plio-Pleistocene when the environment of Madagascar was cooler and drier as this may have facilitated speciation in this group.

It is more difficult to make inferences about speciation between the *F*. *oustaleti* complex and the *F*. *verrucosus* complex because these groups are older and assumptions about the species ancestral ranges are likely not valid [16], but this study provides some insight. Divergence between the *F*. *verrucosus* complex and the *F*. *oustaleti* complex occurred during the Pliocene or Miocene, and niches between the complexes are conserved. While individuals of the *F*. *verrucosus* complex are confined to south and southwest Madagascar, we found several localities where individuals of the two complexes are found syntopic (see Fig 2B). It is unlikely that the watershed hypothesis played a role in diversification between the complexes, since the divergence is dated as older than the Pleistocene and the current distributions of the species show no evidence of restriction to watersheds.

### Are there multiple species nested within *F*. *oustaleti* and *F*. *verrucosus*?

In this study, we identified high mitochondrial diversity, but little nuclear divergence. Nuclear data (CMOS and RAG1) only support the presence of two genetic clusters within the *F*. *verrucosus* complex, and only the very divergent northern clade in the *F*. *oustaleti* complex is supported by nuclear data. In most instances, the program STRUCTURE also recovered four genetic groups using two independent ways to assess K, and these genetic clusters are generally consistent with the clades labeled in Fig 1A. The mitochondrial clades with nuclear support were also well supported in species tree analyses (Fig 2A). However, we were unable to identify any morphological characters that would distinguish the different clades. We have chosen at this time not to recognize additional species due to the low divergences in the nuclear genes (CMOS and RAG1) and the conservative morphological variation. However, we recognize that in the future, faster evolving nuclear genes and additional morphological characters may strengthen the case for dividing these taxa into additional species.

## Conclusions

We found that there are two well-supported clades within both *F*. *oustaleti* and *F*. *verrucosus*, using both mitochondrial and nuclear data. However, there are no clear morphological distinctions between the genetic clades found within each species. We thus hesitate in describing new species at this time, especially since nuclear and morphological support for each clade is low, but note that additional genetic data may strengthen the case for dividing these taxa into additional species.

This study has also clarified the range of the Malagasy giant chameleons. The range of the *F*. *verrucosus* complex is restricted to southern and southwestern Madagascar, and the species complex is found only as far north as the Mangoky River in the southwest. However, the *F*. *oustaleti* complex has a large distribution, ranging as far south as Marolinta and throughout central and northern Madagascar. Additionally, clades within *F*. *verrucosus* have a parapatric distribution, whereas clades within *F*. *oustlaeti* are allopatrically distributed across the Sofia river. Diversification in the *F*. *verrucosus* complex occurred during the Plio-Pleistocene, niche divergence is supported, and the sister clades are parapatrically distributed. In contrast, diversification within *F*. *oustaleti* occurred earlier, either in the Pliocene or Miocene, clades are allopatrically distributed across the Sofia River in Madagascar, and niches between the sister clades are conserved. Divergence within *F*. *verrucosus* is most consistent with patterns expected from ecologically mediated speciation, whereas divergence in *F*. *oustaleti* most strongly matches the patterns expected from the riverine barrier hypothesis.

## Supporting Information

S1 Fig Phylogenetic relationships between the *Furcifer verrucosus* complex, the *Furcifer oustaleti* complex, and near outgroups using partial fragments of the mitochondrial genes ND2 and ND4, reconstructed in the ML tree. (TIF)

S2 Fig Maximum likelihood (raxML) tree recovered using both mitochondrial and nuclear data. Full sample names are provided. The tree topology is congruent with the one found using mitochondrial data alone (see Fig 1 and S1 Fig).
(TIF)

S3 Fig Species tree analysis with divergence dating using ND2 rate of 0.65%. All divergence times are indicated with an arrow; posterior support values are below branches.
(TIF)

S4 Fig Species tree analysis with divergences, when both the fossil and the biogeographic calibrations are applied. Divergence times are indicated with an arrow, and poster support values are below branches. Note that divergence dates do not vary with respect to those in Fig 2, in regards to the geologic period when diversification occurred.
(TIF)

S1 Table Localities, sample numbers, coordinates, and Genbank accession numbers for all samples included in this study. (XLSX)

S2 Table The results of the niche identity test implemented in ENMTools. Each excel tab showing comparisons made in Fig 3 (a vs. b = *Furcifer verrucosus*; c vs. d = *Furcifer oustaleti;* and verr vs. oust. *= Furcifer oustaleti* and *Furcifer oustaleti*.)
(XLSX)

S3 Table The results of the niche background test implemented in ENMTools. Each excel tab showing comparisons made in Fig 3. Same labels as in S2 Table.
(XLSX)

S4 Table Morphological results for all specimen examined in this study. (XLSX)
