# Supplementary figures and images for: A Phylogeographic Assessment of the Malagasy Giant Chameleons (Furcifer verrucosus and Furcifer oustaleti)
Source: PLoS One. 2016 Jun 3;11(6):e0154144. doi: 10.1371/journal.pone.0154144 (PMC4892496; doi:10.1371/journal.pone.0154144)

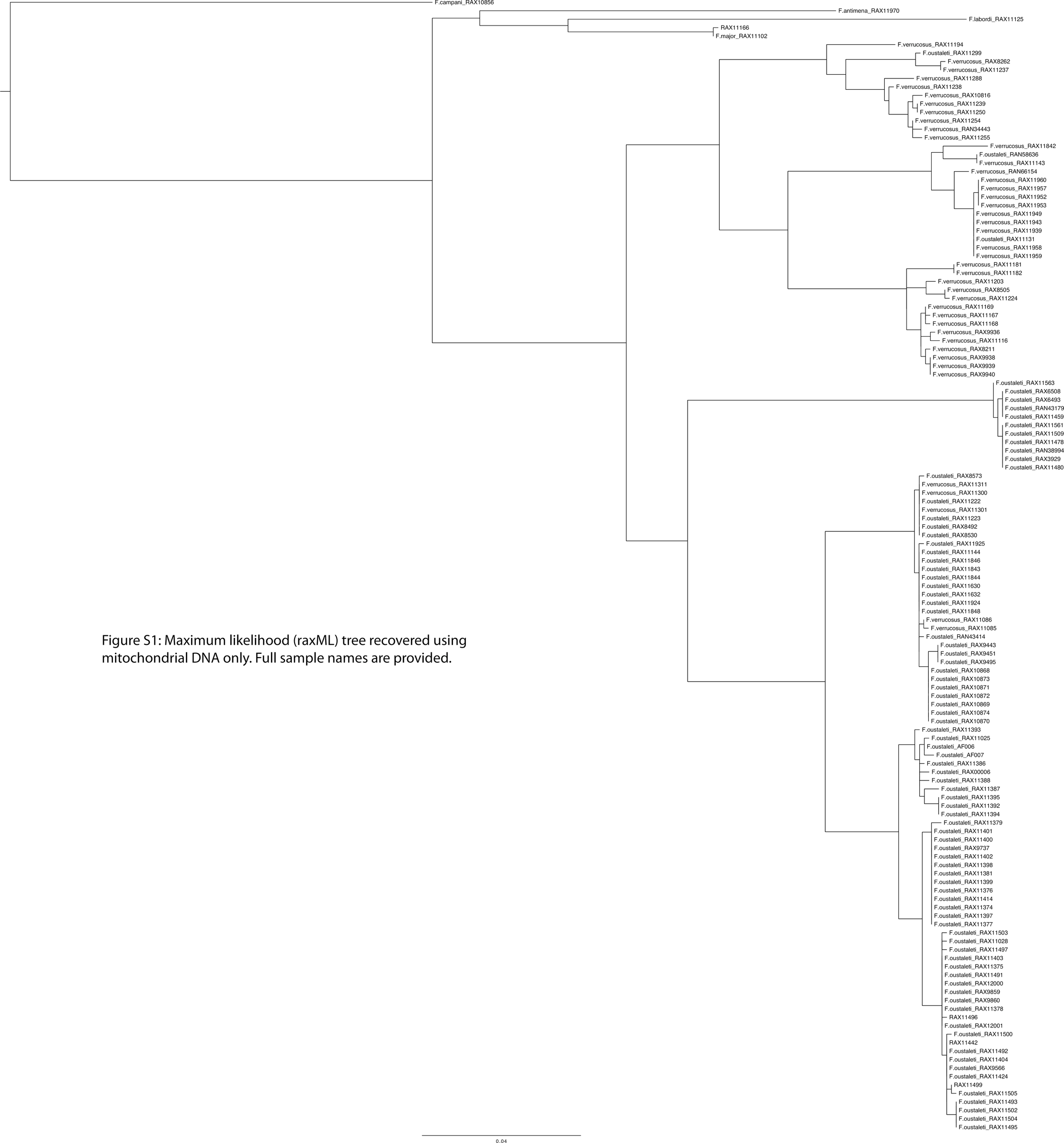

Supplement: S1 Fig — (TIF) [file pone.0154144.s001.tif]

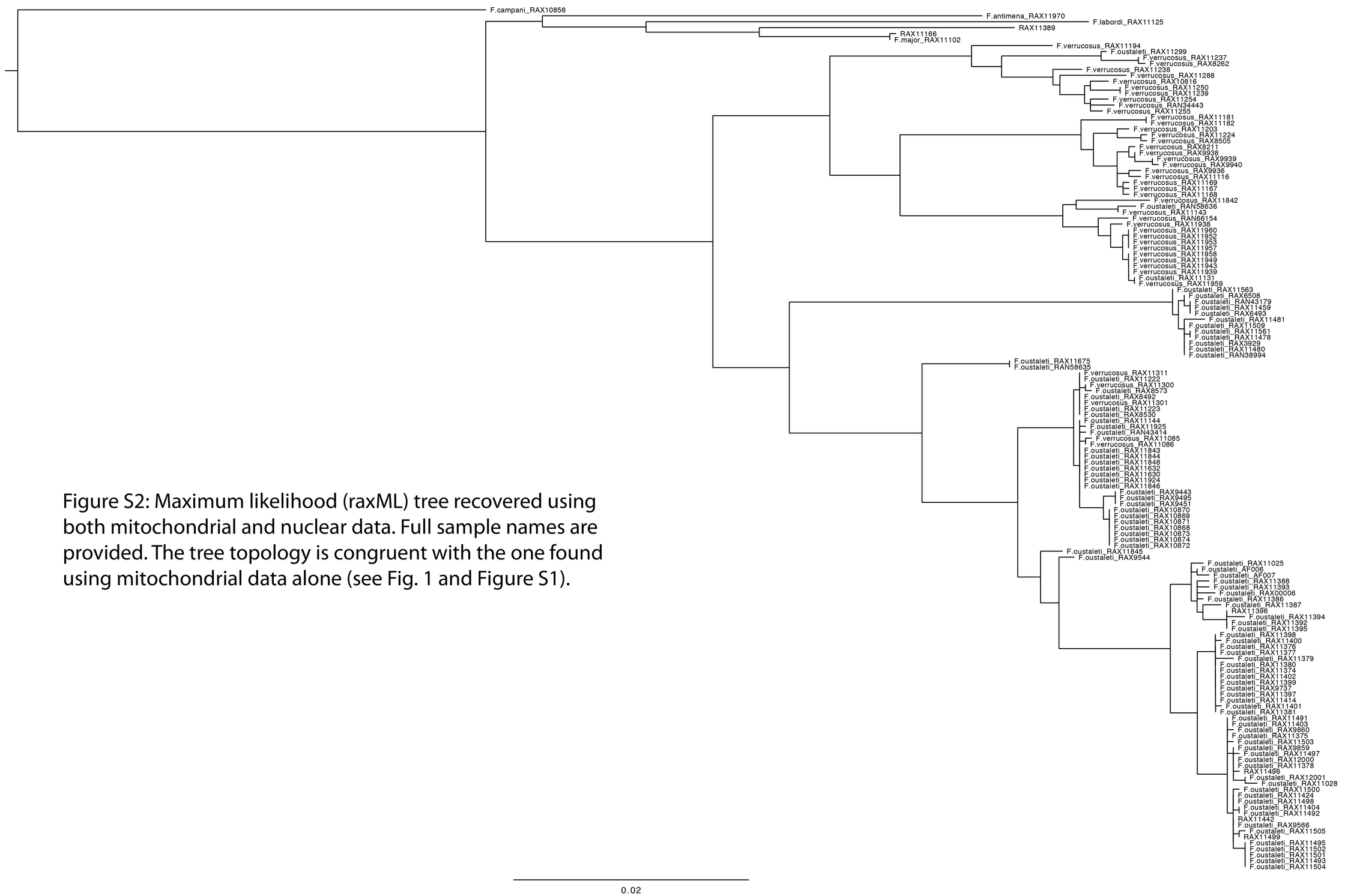

Supplement: S2 Fig — Full sample names are provided. The tree topology is congruent with the one found using mitochondrial data alone (see Fig 1 and S1 Fig). (TIF) [file pone.0154144.s002.tif]

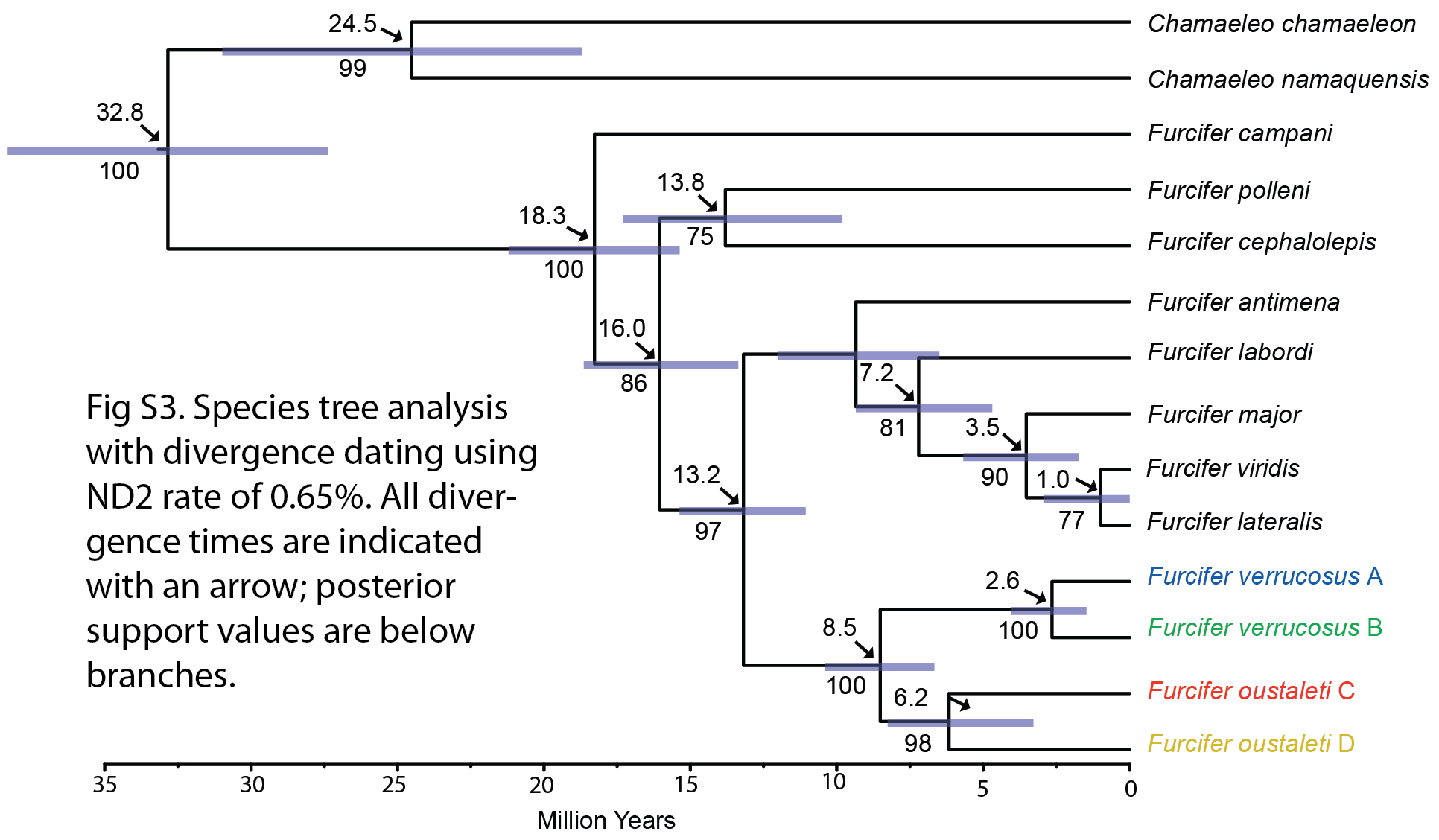

Supplement: S3 Fig — All divergence times are indicated with an arrow; posterior support values are below branches. (TIF) [file pone.0154144.s003.tif]

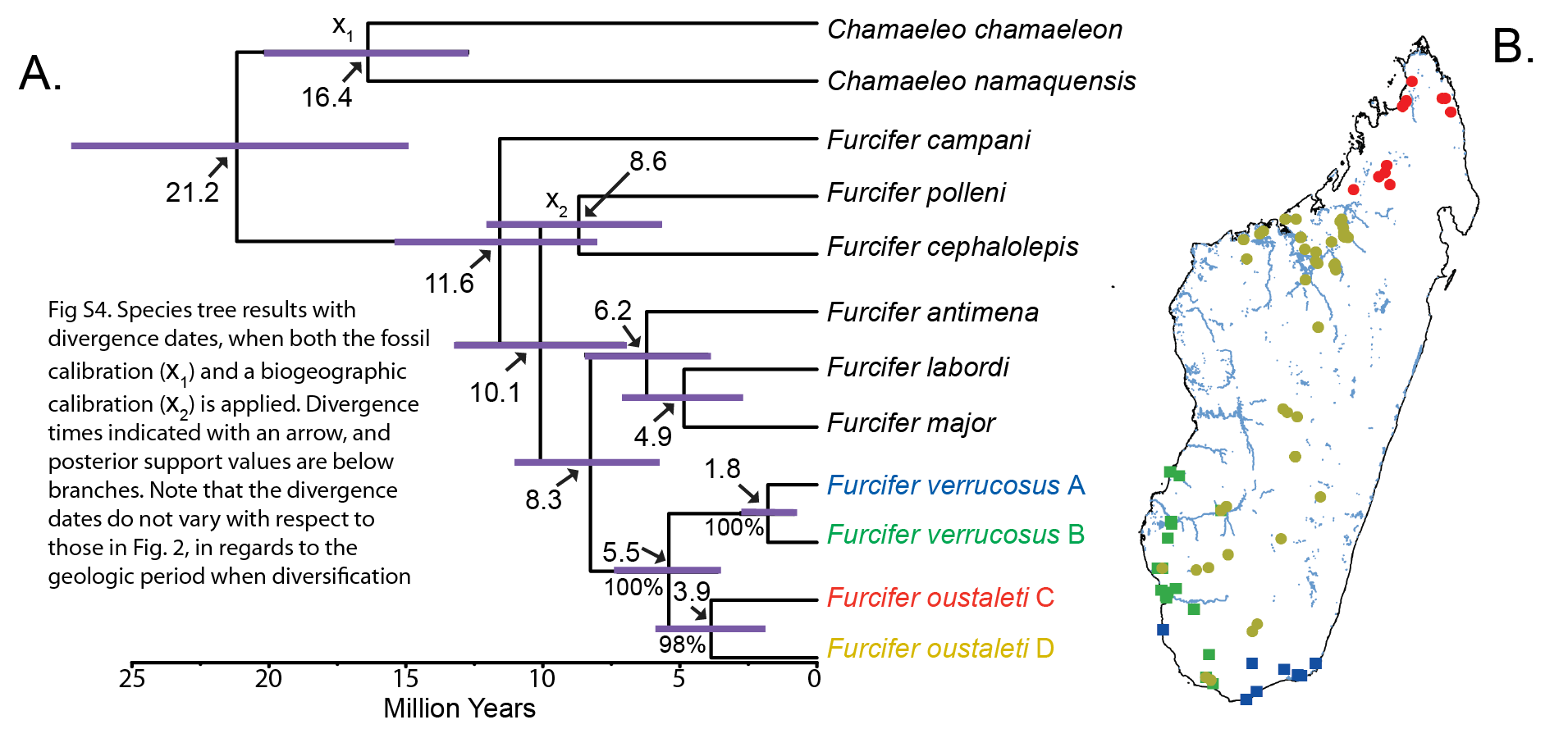

Supplement: S4 Fig — Divergence times are indicated with an arrow, and poster support values are below branches. Note that divergence dates do not vary with respect to those in Fig 2, in regards to the geologic period when diversification occurred. (TIF) [file pone.0154144.s004.tif]
